# Immune response to zinc oxide inhalation in metal fume fever, and the possible role of IL-17f

**DOI:** 10.1038/s41598-023-49430-5

**Published:** 2023-12-14

**Authors:** Éva Szűcs-Somlyó, József Lehel, Kornél Májlinger, Fruzsina Tóth, Ákos Jerzsele, Csaba Kővágó

**Affiliations:** 1 Department of Epidemiology and Infectious Diseases, University of Veterinary Medicine, Istvan str. 2., 1078 Budapest, Hungary; 2 Department of Food Hygiene, University of Veterinary Medicine, Istvan str. 2., 1078 Budapest, Hungary; 3 Department of Materials Science and Engineering, Faculty of Mechanical Engineering, Budapest University of Technology and Economics, Bertalan Lajos str. 7., 1111 Budapest, Hungary; 4 MTA-BME Lendület Composite Research Group, Bertalan Lajos str. 7., 1111 Budapest, Hungary; 5 University of Veterinary Medicine, Istvan str. 2., 1078 Budapest, Hungary; 6 Department of Pharmacology and Toxicology, University of Veterinary Medicine, Istvan Str. 2., 1078 Budapest, Hungary; 7 National Laboratory of Infectious Animal Diseases, Antimicrobial Resistance, Veterinary Public Health and Food Chain Safety, University of Veterinary Medicine H-1078, Istvan str. 2., Budapest, Hungary

**Keywords:** Experimental models of disease, Immunopathogenesis, Inflammation, Metals, Cytokines

## Abstract

Metal fume fever (MFF) is a work-related disease caused by the inhalation of metal particles, including zinc oxide. Chronic asthma may develop as a long-term consequence of exposure, particularly for welders and metal workers who are most at risk. In this study, we investigated the effects of ZnO fume inhalation on multiple inflammation-related cytokine- and cytokine receptor genes in mice from lung and lymph node samples, to explore the role of these in the pathogenesis of MFF. In our experiments, the animals were treated with a sub-toxic amount of ZnO fume for 4 h a day for 3 consecutive days. Sampling occurred 3 and 12 h post-treatment. We are the first to demonstrate that ZnO inhalation causes extremely increased levels of IL-17f gene expression at both sampling time points, in addition to increased gene expression rates of several other interleukins and cytokines, such as IL-4, IL-13, CXCL5, CSF-3, and IFN-γ. Our animal experiment provides new insights into the immunological processes of early metal fume fever development. IL-17f plays a crucial role in connecting immunological and oxidative stress events. The increased levels of IL-4 and IL-13 cytokines may explain the development of long-term allergic asthma after exposure to ZnO nanoparticles, which is well-known among welders, smelters, and metal workers.

## Introduction

Metal fume fever (MFF), which can be realized after a few days without exposure correlated with weekend rest, is a well-known work-related disease caused by inhalation of biogenic metals and metal oxides. Despite of the fact that the orally uptake of these metals is strictly regulated, the inhalation of them can cause inflammation in various organs. Zinc oxide is one such metal oxide that can cause specific symptoms in humans. This disease is called by various names, e. g. foundry fever, brass chills, spelter shakes, depending on the specific occupation (smelter, welder etc.)^1–3^, depending on the specific occupation (smelter, welder, etc.). The disease is also known as "Monday morning fever" because symptoms often appear on Monday morning after a few days without exposure correlated with weekend rest^4,5^.

Metal fume fever can be induced not only by zinc but also by other metals such as copper, manganese, cadmium, mercury, and magnesium in combination^1,4^. Welders and metal workers are the most exposed to this disease, and its prevalence can reach 35% among welders due to repeated exposure, which may indicate a genetic predisposing factor for the development of the disease^6,7^. The disease may also be recurrent, with reports of individuals suffering more than 10 cases of metal fume fever^4,8,9^. Workers are exposed not only to zinc chills but also to other serious harms due to their exposure to welding fumes, smoke and dust from smoking and other sources such as respiratory impairment, pulmonary fibrosis, siderosis, deposition of various metal particles in the lungs, and lung cancer^10–13^. Generally, the symptoms of MFF begin within hours of metal inhalation and include a sweet, metallic taste in the mouth, throat irritation, and breathlessness followed by flu-like respiratory symptoms (dry cough, difficulty breathing), furthermore fever, muscle aches, chills, fatigue, and malaise, or even vomiting and headache. The creation of the correct diagnosis is difficult due to the non-specific symptoms, thus the treatment is only symptomatic including bed rest, pain, and fever relief^1,4^. Beside the typical symptoms, allergic symptoms, urticaria, and angioedema, chronic asthma can be manifested due to the repeated exposure to zinc oxide^2,14–16^.

Nanoparticles (NPs) smaller than 100 nm have a high mass/surface area ratio, making their biological activity more dependent on their physical properties than on their chemical compositions. Therefore, it is possible that inhalation of different metals may induce similar symptoms^17–19^. Particles smaller than 5 microns can enter the alveoli and cannot be filtered out of the airways by cilia. Metal oxide particles smaller than this, between 20 and 3000 nm^20,21^, are produced from any welding and pose a danger to workers^22^. Hence, many countries have set an occupational exposure limit for ZnO of 5 mg/m^3^ for 8 h of work^1,4,7,23–25^.

However, symptoms of metal fume fever can develop even at inhaling sub-toxic concentrations, and inhalation of lower concentrations of zinc oxide can also cause disease. Monsé et al. (2018) detected increased level of C reactive protein (CRP) and serum amyloid A (SAA) in the blood after inhalation of 1 mg/m^3^ of zinc oxide, and a fever of 39.5°Cwas measured after inhalation of 2 mg/m^3^ of zinc oxide^26^. This level of exposure can lead to enhance a three-fold higher risk of cardiovascular disease, including myocardial infarction and stroke in long term^24^. Several studies have been suggested that the existing health limits for occupational exposure to ZnO should be revised^24,26,27^.

The exact pathophysiology of MFF is still unclear, but several studies suggest that it involves an inflammatory process that causes the clinical symptoms. Exposure to sub-toxic concentrations of ZnO has been shown to increase endogenous pyrogens such as tumor necrosis factor-α (TNF-α), interleukin (IL) IL-1, IL-6, which are involved in the development of fever, and acute phase proteins SAA, CRP, in both serum and broncho-alveolar lavage fluid (BALF)^7,24,26,28–30^. Polymorpho-nuclear (PMN) cells are also elevated in blood^16,31,32^. Researchers are still trying to determine what triggers the immune system during the initial phase of disease development after inhalation of an essential metal.

Several studies suggest that ZnO particles are transported into the lysosomes of immune cells. In the lysosomes' acidic environment, these particles ionize, compromising lysosomal integrity and triggering oxidative stress through the release of reactive oxygen species (ROS). ROS can initiate an inflammatory cascade and lead to cell death^28,33^. To counteract this, the body's physiological antioxidant enzyme system, including superoxide dismutase (SOD) and catalase (CAT), provides cellular protection. When oxidative stress overwhelms this system, the nuclear factor kappa B (NF-κB) signaling pathway activates, producing pro-inflammatory cytokines like TNF-α, IL-1, and IL-6, or leading to cell apoptosis or necrosis under severe stress^7,11,34,35^. Glutathione (GSH) and metallothionein (MT), known for binding metal ions with their "thiol" group, play central roles in defense against toxic metal ions^36,37^.

Other researchers propose that metal oxides act as haptens, binding to proteins to form complete antigens. This process could lead to a late form of type I hypersensitivity reaction, marked by increased serum immunoglobulin E (IgE) levels. Such reactions have been observed in patients with MFF, exhibiting allergic symptoms like urticaria, persistent pruritus, and angioedema^2,28,38,39^.

In a long-term study, 39.2% of the 286 welders showed symptoms of MFF and 13.8% developed welding-related asthma on average during the 15-month follow-up period. At the end of the study, a Prick test showed 11.8% positivity for metals associated with MFF, such as Cu, Zn, Cr, Mn^6^. Several studies have confirmed increased levels of IgE after Zn exposure^2,28^.

In an experiment, researchers observed neutrophil granulocyte (Ne) infiltration and eosinophil granulocyte (Eo) activation, as well as elevation of IL-5, IL-13, TNF-α, and IFN-γ cytokines in BALF after sub-toxic ZnO exposure in mice^29^. However, subsequent experiments investigating the presence of cytokines associated with allergic reactions in BALF were negative^40,41^.

Type I hypersensitivity reactions result from IgE-mediated interactions with mast cells, leading to the degranulation of eosinophilic and basophilic cells. This reaction does not manifest during the initial allergen exposure but occurs after a sensitization phase. Initially, the allergen activates the humoral response, producing IgM antibodies. Under the influence of cytokines like IL-4 and IL-13, cells transition towards IgE, while IL-3 and IL-5 activate eosinophilic granulocytes. Upon subsequent allergen exposures, mast cells release histamine and other proteases, leading to a secondary, slower hypersensitivity reaction. Key enzymes, including matrix metalloproteinase (MMP), induce inflammation with notably elevated MMP-9 levels observed during MFF incidents. The MAPK pathway, when activated by certain stimuli, produces pro-inflammatory cytokines, triggering fever. Allergies, categorized as type I hypersensitivity, can be caused by various sources, including metals like zinc oxide. Symptoms range from severe anaphylaxis to milder local reactions. Some allergic responses are similar to those of metal fume fever, as documented in research^26,38^.

## Results

### ZnO fume generation and fume concentration control

**Figure 1:**
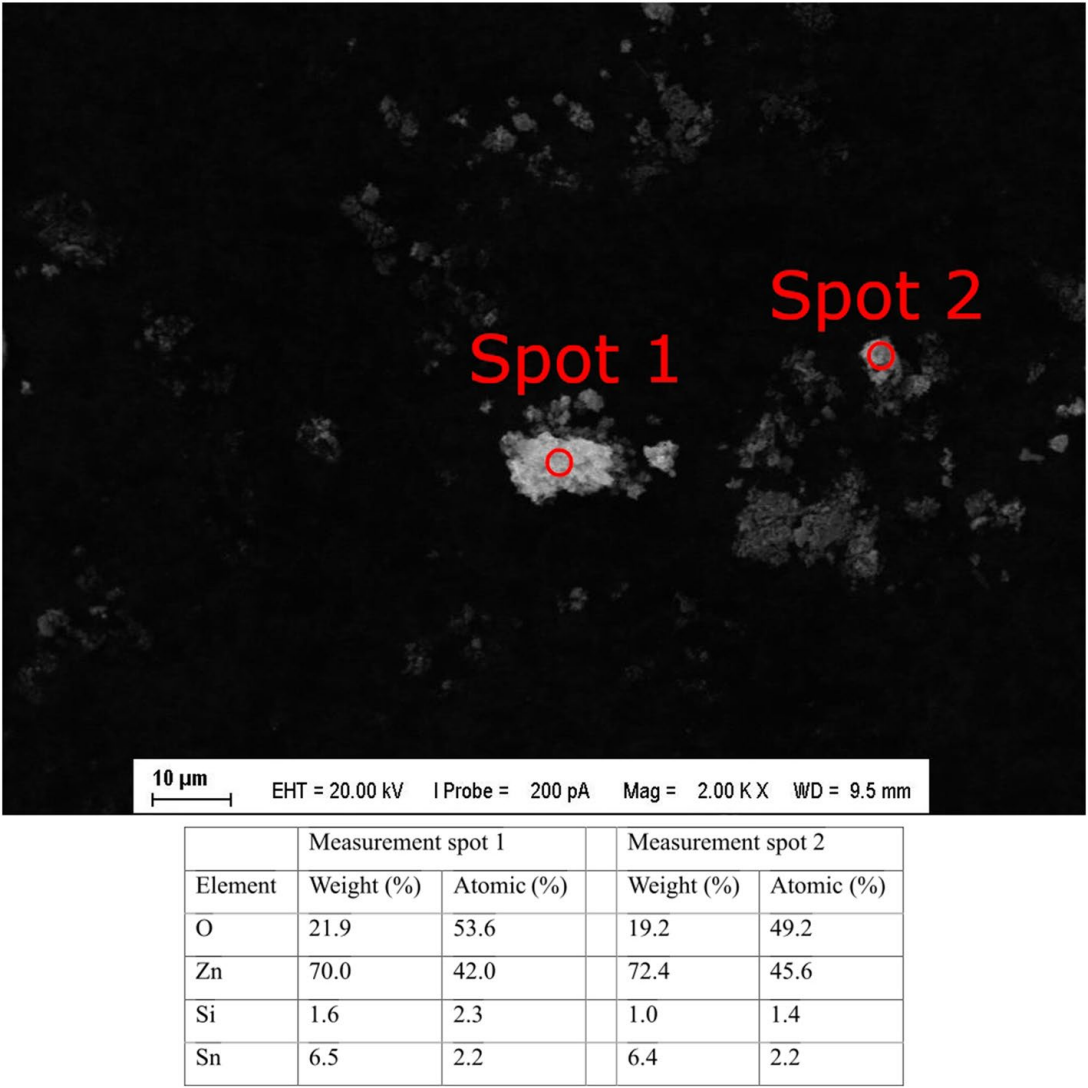
SEM image of the sampled, ZnO polluted air. The brighter particles are conglomerates of ZnO particle; the chemical composition data measured in the designated spots of them are shown in the table.

Examination by scanning electron microscopy (SEM) showed that the size of zinc oxide nanoparticles was approximately 10–20 nm, and some of these formed conglomerates ranging in size from 500 to 3000 nm. Energy dispersive spectroscopy (EDS) conducted in parallel showed that the chemical composition of the examined particles matched the theoretical composition of ZnO with very little contamination. The SEM image and EDS measurement results are shown in Fig. 1.

**Figure 2:**
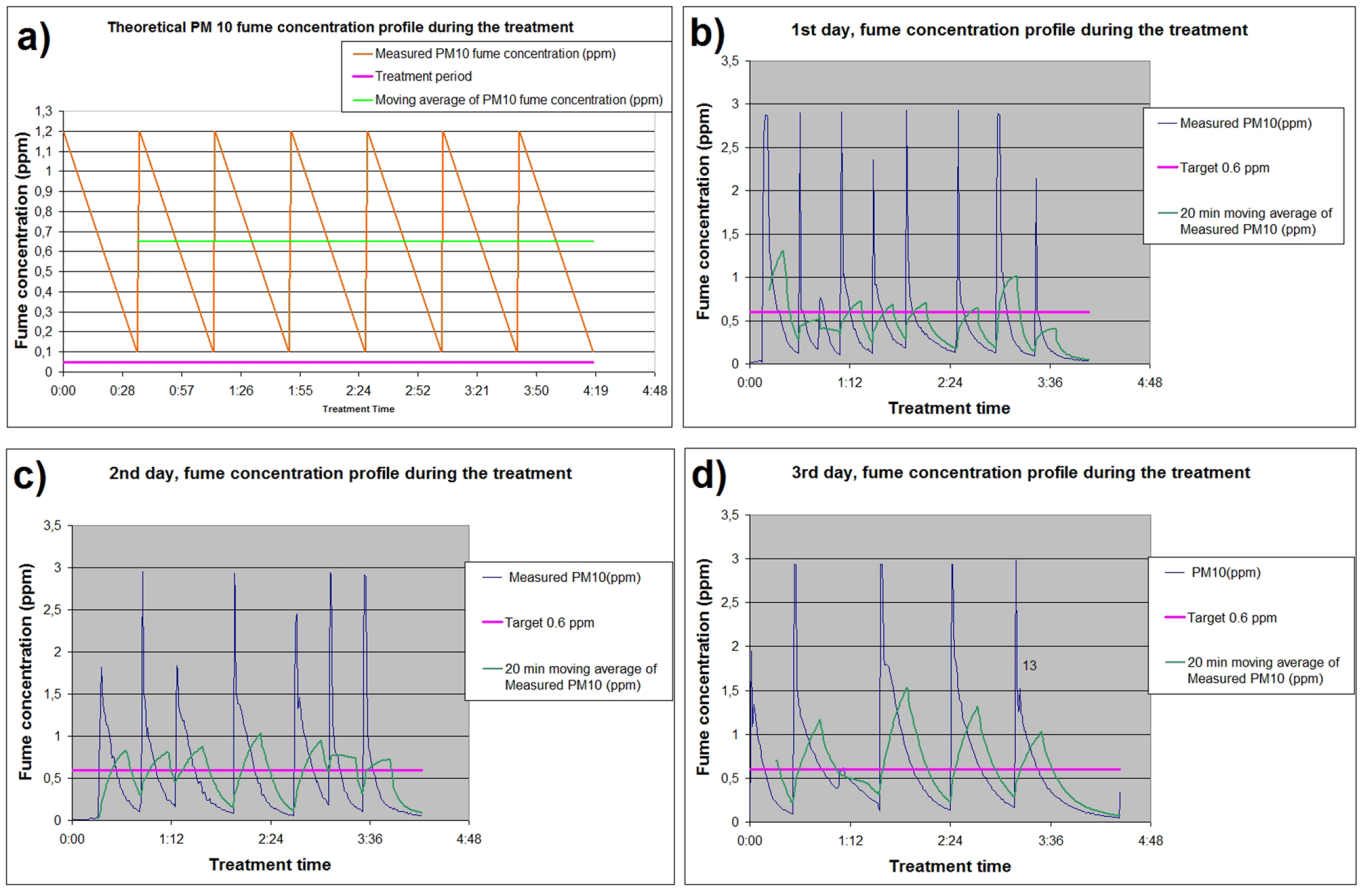
The theoretical air concentration regime of the PM10-sized ZnO particles during the 4-h treatment ppm versus the measured experimental results. The mean target concentration was 0.6 ppm in every case (purple line). (**a**) shows the planned theoretical ZnO concentration profile, while (**b**–**d**) show the actual measured data during the 3-day treatment period.

The control measurement of the fume concentration showed that we generally achieved the target mean of approximately 0.6 ppm fume concentration in the treatment chamber. The final mean fume concentration, calculated from all the data collected during the complete 3-day treatment period, was 0.53 ppm, equivalent to 1.76 mg/m^3^. The measured air concentration diagrams can be seen in Fig. 2.

Parallel, we have measured the PM 2.5 concentrations as well. We obtained the results that in average, the PM2.5 particle concentrations are 2/3–3/4 of the PM10 particle concentrations in the same treatment (more details are to be found in the Additional information section, Figure S2).

### Pretreatment and selection of susceptible individuals by measuring locomotor activity

During the series of pretreatments, we treated three groups of four animals, for a total of 12 animals. Using the described method, we were able to separate four animals that showed decreased locomotor activity between 36 and 48 h post-treatment time, compared to the other treated animals.

### Gene expression measurement by quantitative real-time PCR

**Figure 3:**
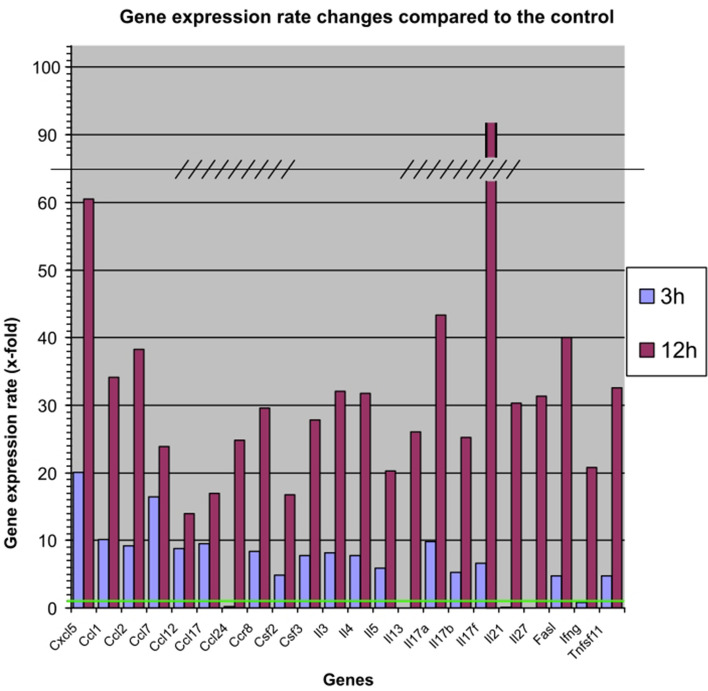
Gene expression results of the experiment. The green line represents the gene expression rate 1, which is the control value. Results were generated from lung and mediastinal lymph node samples pooled together.

The most important gene expression rate measurement data are shown in Fig. 3. The complete dataset can be seen in Additional information, Table S2.

Among all the studied inflammatory parameters, IL-17 subfamily gene expression rates had the highest gene expression rate. IL-17a showed a 44-fold increase, IL-17b a 25-fold increase, and IL-17f a 92-fold increase in gene expression at 12 h after the end of treatment. Among the cytokines, gene expression levels of IL-3, IL-4, IL-13, IL-21, and IL-27 increased about 30-fold by hour 12. Similarly, IFNγ (showed as Ifng in the graph) and IL-5 gene expression rates increased about 20-fold of the control level at hour 12.

At 3 h after the end of exposure, most cytokine gene expression levels remained below the tenfold threshold value, with a few outliers. At hour 3, the highest increase (20-fold) was observed for the alpha chemokine, Cxcl5, which increased to 60-fold by hour 12. The next highest increase (17-fold) was observed for the beta-chemokine, Ccl7, which increased 24-fold by hour 12. Several beta-chemokines, such as Ccl1, Ccl2, Ccl12, Ccl17, and chemokine receptor rate Ccr8, increased 8–tenfold, and these parameters further increased by hour 12, with Ccl1, Ccl2, and Ccr8 exceeding 30-fold at the second measurement.

At the 3rd hour of measurement, some parameters showed a strong down-regulation. IL-27 was the lowest at 0.05-fold, IL-21 at 0.11-fold, and Ccl24 (eotaxin 2) at 0.17-fold. However, these cytokines showed a 25–31-fold up-regulation at the 12th hour measurement.

The gene expression rate of Fasl showed a prominent increase at hour 12 (40-fold). Among members of the tumor necrosis factor family, Tnfsf11 showed the highest increase, with a 30-fold jump in gene expression at hour 12. The other members of the TNF family studied were all elevated at different rates of fourfold to 33-fold. Of the colony-stimulating factors, Csf2 increased up to 17-fold and Csf3 up to 29-fold at hour 12. The expression of the other cytokines and chemokines showed more moderate increases.

## Discussion

Our ZnO fume EDS and PM measurements, in comparison to ambient air data (Additional information, Figure S2), indicate that particle concentrations during treatments were significantly higher, more than a magnitude above ambient levels. This suggests that the majority of particles in the treatment chamber originated from the ZnO fume we generated. Further, parallel PM10 and PM2.5 measurements closely aligned, with daily averages nearly identical. This implies that most ZnO fume particles fall within the PM2.5 size range, capable of reaching the alveoli in experimental animals, affirming the suitability of our experimental setup.

Our study marks the first in international literature to investigate MFF involving mediastinal lymph node samples. Contrary to the prevalent focus on lungs in MFF pathomechanism, our results, alongside previous hypotheses, suggest the involvement of blood in this process^42,43^. Evaluating the detectability of target mRNAs in both lung-only and pooled lung + mediastinal lymph node samples revealed higher detection of inflammation-related gene mRNA in the pooled samples (Figure S3, Additional information). This informed our decision to use pooled samples for higher accuracy in final results.

In exploring MFF's pathomechanism, we analyzed mRNA gene expression levels of various cytokines, vital in regulating immune responses. They influence white blood cell mobility during infection and inflammation and are produced by diverse cell types, often with pleiotropic effects. We focused on specific inflammatory factors exhibiting significant results and having synergistic or antagonistic relationships in immune regulation.

Before analyzing our data, it is crucial to assess the validity of our results in comparison with historical data. We validated our experiment by comparing our findings to those from recent studies, as detailed in Table 1. Most non-human studies used intratracheal instillation of ZnO-containing fluid in rodents, primarily mice, with bronchoalveolar lavage fluid (BALF) being the primary sample for investigation. In contrast, our study involved unrestrained inhalation experiments with freshly made ZnO fume-containing air, utilizing lung and mediastinal lymph nodes for analysis. Despite these methodological differences, our comparison with previous data identified several commonalities in the datasets examined. Given that protein translation from mRNA typically takes about 12 h, our mRNA results at 12 h post-treatment align with protein measurement data obtained at 24 h post-treatment in earlier studies. Furthermore, like many of these studies, our experiment also involved a small number of animals^28,29,34,41,42^. Overall, our results are credible and valid despite the limited number of animals used, and we ensured compliance with the 3R principles for animal experimentation.

**Table 1:** Comparison of our results with historical data.

Own results	Historical data	References
IL-13 gene expression rate 26-fold, Ccl-24 (eotaxin-2) expression rate ~ 25-fold in 12 h	Eotaxin, IL-13 level increase in 24 h in BALF	^28^
CSF3 expression rate 28-fold in 12 h	CSF3 level increase in 24 h in BALF	^41^
Cxcl-1 ~ 34-fold, Ccl-2 38-fold, CSF2 16-fold gene expression rate increase in 12 h	Cxcl-1, Ccl-2, CSF2 level increase in BALF in 24 h	^34^
IL-5 20-fold, IL-13 26-fold, IFNγ 20-fold gene expression increase in 12 h	IL-5, IL-13, IFNγ level increase in BALF in 24 h	^29^
IL-13 26-fold, IL-4 32-fold gene expression increase in 12 h	IL-4, IL-13 level increase in whole blood assay in 24 h	^42^

In our preliminary experiment designed to identify animals sensitive to metal fume fever (MFF), we observed a 30% incidence rate of sensitivity, which is consistent with the 30–35% sensitivity rate in humans reported in different studies^6,7^. Our gene expression research primarily focused on Colony Stimulating Factors (CSF) mRNA, providing valuable insight into bone marrow cell production during MFF. We found a significant upregulation in CSF3 (also known as G-CSF), indicating enhanced granulocyte maturation. Conversely, while CSF1 levels remained low, CSF2 levels rose above the threshold by the 12th hour. Furthermore, there was a notable increase in IL-3 and IL-4, with the latter particularly influential in mast cell differentiation, as noted in references 44–46^44–46^.

In our study, the chemokine Cxcl5 demonstrated significant increases in expression, signaling the onset of neutrophil granulocyte-driven local inflammation in the early stages of metal fume fever (MFF). Cxcl5 primarily directs neutrophils to sites of injury, thereby activating the MAPK pathway, which underscores the critical role of lung-specific alveolar cells in this process.

The results from our interleukin analysis were particularly revealing, indicating inflammation activities not only by neutrophilic but also by eosinophilic and basophilic granulocytes. IL-4, known for its influence on B cell differentiation and antibody production, was upregulated in a manner similar to that observed in inflammatory respiratory conditions like asthma. Additionally, IL-5, which fosters eosinophilic granulocyte maturation, showed a significant increase, accompanied by an elevation in Ccl24. The roles of IL-4 and IL-13 in facilitating the isotype switching of antibodies to IgE point towards their association with allergic reactions. While the immuno-allergic theory behind MFF is not widely accepted, our findings support the hypothesis that hypersensitivity reactions may contribute to the pathomechanism of MFF, as suggested in the literature^38^.

Recent findings in our research revealed that IL-17f gene expression experienced the most significant up-regulation, along with notable increases in IL-17a and IL-17b genes at both measured time points. A key function of IL-17f includes stimulating acute neutrophilic inflammation by inducing various pro-inflammatory cytokines and chemokines such as CSF3, CSF2, Cxcl1, Cxcl5, Ccl2, and Ccl7, aligning closely with our results^47^. Th17 cells, often referred to as inflammatory T helpers, are typically produced in response to pro-inflammatory cytokines. Interestingly, the IL-17 b, c, d subfamily is not produced by lymphocytes^47^. The IL-17f subfamily's association with oxidative stress-induced inflammation, previously studied in relation to mercury and cadmium, suggests a potential similar process with zinc. This is particularly relevant as MFF is known to be associated not only with zinc but also with other metals like cadmium^48^.

Previous studies have shown that IL-17f upregulation may be induced by cytotoxic or apoptotic events, with oxidative stress believed to initiate the upregulation of this cytokine^49^. Currently, the prevailing theory in MFF pathogenesis is that of oxidative stress-induced inflammation. In cases of oxidative stress, the immune reaction is primarily initiated by cell necrosis, occurring through damage-associated molecular patterns (DAMP) or via the MAPK pathway.

Currently, the most accepted theory in the pathogenesis of metal fume fever (MFF) is inflammation induced by oxidative stress. This immune reaction is typically triggered by cell necrosis via damage-associated molecular patterns (DAMP) or the MAPK pathway. However, in MFF cases, significant tissue damage that could activate fever through the DAMP pathway is not commonly reported, and the MAPK pathway does not typically modulate the IL-17 subfamily. Therefore, a plausible explanation for our findings is that oxidative stress directly induces a substantial increase in IL-17f, leading to acute inflammation and fever development. The marked up-regulation of IL-17f at both 3 and 12 h post-treatment in our study is a crucial finding, linking oxidative stress response directly to immunological processes in MFF's pathomechanism.

IL-21 was greatly down-regulated at 3 h post-treatment but significantly up-regulated at 12 h. While IL-21 is an autocrine factor maintaining IL-17a and f production, it can also limit inflammatory processes by up-regulating IL-10. Similarly, an increase in IL-27 gene expression helps counteract inflammation, contributing to the self-limiting nature of MFF.

Our results confirm that inhaling zinc oxide triggers an acute inflammatory response, characterized by increased levels of IL-3, IL-4, IL-5, and IL-13, along with an IL-17-mediated neutrophilic granulocytic response. These cytokines may contribute to the development of type I hypersensitivity. Despite continued high levels of pro-inflammatory gene expression at 12 h post-exposure, a 32-fold increase in IL-27 indicates the activation of regulatory T cells, suggesting a potential for spontaneous recovery from MFF.

## Conclusion, proposed pathway

Our animal study provided comprehensive insights into the early immunological processes of metal fume fever (MFF) development. We successfully measured gene expression rates of various cytokines and their receptors, notably in the mediastinal lymph nodes, an area previously unexamined in MFF research. This study is the first to document changes in gene expression of these cytokines and receptors during MFF. Our findings particularly underscore the importance of IL-17f in bridging immunological and oxidative stress events in MFF.

**Figure 4:**
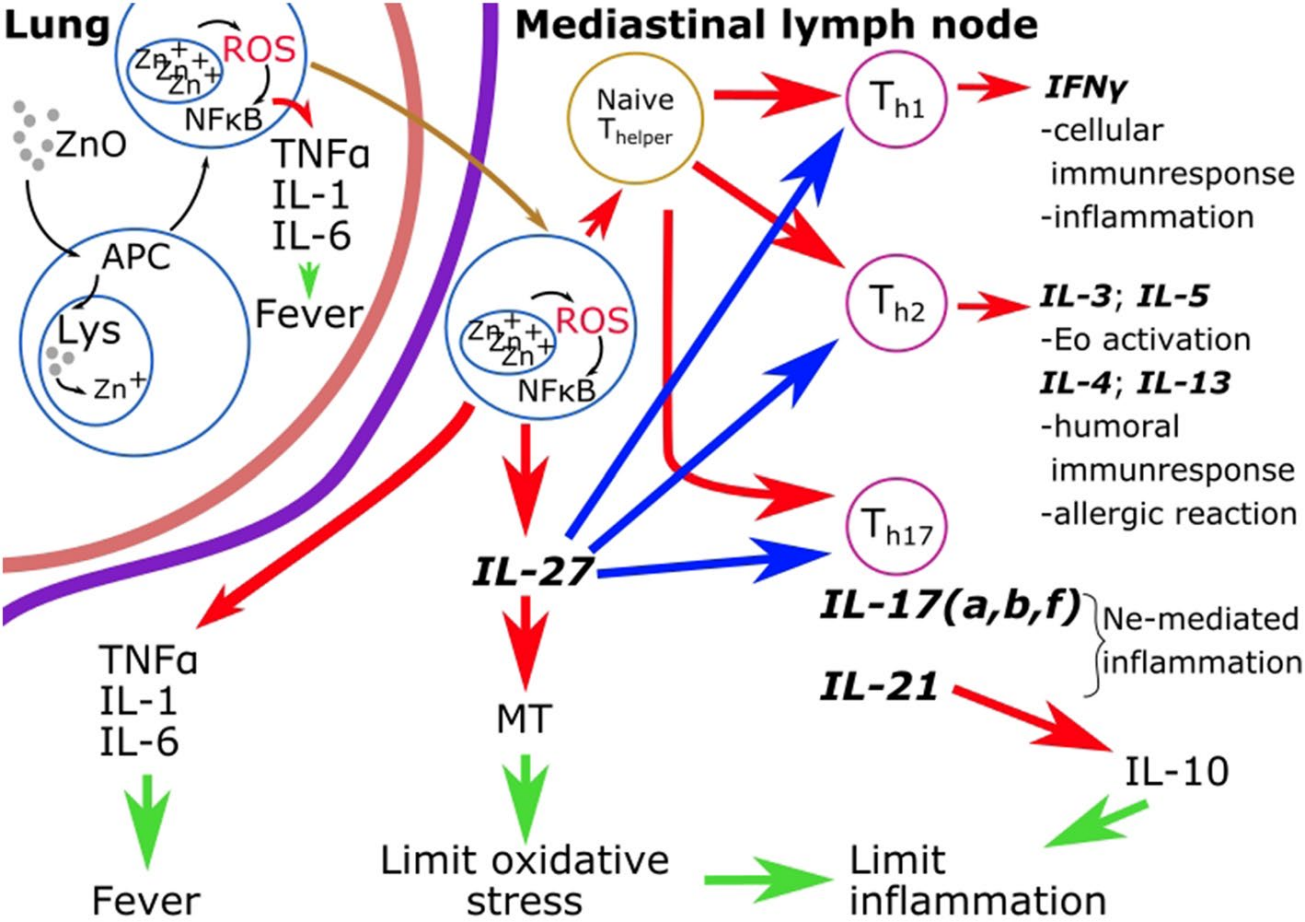
The proposed pathomechanism of metal fume fever based on our experimental results and previous data. Red arrows indicate activation/production pathways, blue arrows represent inhibition pathways, and green arrows indicate the results of the processes. Cytokine gene expression rates written in bold/italic were measured in our experiment.

The role of IL-17f allows for a synthesis of the two competing hypotheses regarding MFF's pathomechanism. Our data delineate the following immunological events post-inhalation of ZnO nanoparticles (ZnONPs), as depicted in Fig. 4.

ZnONPs, due to their small size, can penetrate deep into the lungs, reaching the alveoli where they are predominantly engulfed by alveolar macrophages. This leads to the formation of reactive oxygen species as a result of high Zn + ion concentrations in the lysosomes, subsequently causing elevated oxidative stress and activating the NFκB pathway, as indicated by the dissolution of ZnO particles^49^. Consequently, macrophages begin to produce pro-inflammatory cytokines like TNFα, IL-1, and IL-6. Moreover, macrophages and dendritic cells (DC) travel to nearby lymph nodes to present antigens to naive T helper cells, with antigen-presenting cells (APC) guiding the differentiation of naive Th cells during the immunological synapse.

Activation of the NFκB pathway triggers diverse immune responses. Firstly, it enhances cellular immunity by facilitating Th1 differentiation, which in turn produces IFNγ, an increase of which was observed in our study. Additionally, NFκB activation can lead to the development of Th17 cells, contributing to neutrophil-mediated inflammation. Our study's most significant finding was the elevation of IL-17, alongside increased production of IL-21.

Conversely, NFκB activation can also stimulate humoral immunity by promoting Th2 cell formation. Th2 cells produce IL-3 and IL-5, crucial for activating eosinophil granulocytes. Elevated gene expression levels of IL-4 and IL-13, detected in our study, might explain the development of long-term allergic asthma often observed in humans after prolonged exposure to welding fumes or ZnO inhalation. As these processes predominantly occur in lymph nodes rather than lungs, many previous studies using bronchoalveolar lavage fluid (BALF) as the sample did not detect them. However, activated Th cells also migrate from mediastinal lymph nodes, facilitating the measurement of IL-4 and IL-13 protein level elevations in BALF, as shown in Huang et al.'s 2019 study. Despite this, investigations into eosinophilia and allergic pathogenesis following ZnO exposure have produced inconsistent results, with several studies failing to identify hypersensitivity markers in the blood^40–42^.

In our study, we noted an initial decrease in IL-27 gene expression, followed by an increase at the 12th hour. While IL-27 boosts IFNγ production and cellular immunity, it also augments IL-21 production. This leads to a rise in IL-10 and the number of regulatory T cells, potentially inhibiting Th1, Th2, and Th17 functions and consequently dampening inflammation. Furthermore, IL-27 enhances metallothionein (MT) synthesis, neutralizing free Zn^+^ ions and mitigating oxidative stress. This supports the observation that metal fume fever symptoms usually resolve spontaneously within days^50^. Besides activating IL-21, IL-27 also contributes to long-term inflammation inhibition.

Our experimental outcomes offer insights into why past research on metal fume fever has yielded mixed results. Most data were derived from BALF samples, not accounting for cytokines predominantly produced in local lymph nodes. Additionally, animal studies often lacked pre-screening for MFF sensitivity, leading to varying cytokine profiles between sensitive and non-sensitive animals. Previous studies typically employed intratracheal instillation, where ZnO particles were suspended in liquid, potentially altering their surface characteristics and biological effects. Our methodology was designed to circumvent these factors for more reliable in vivo results. Despite our study's small sample size, the correlation with existing literature and consistent gene expression patterns in key interleukins and chemokines support our findings. While our research provides a detailed view of the initial immunological mechanisms in metal fume fever, further studies are required to fully elucidate the disease's regulatory pathways.

## Material and methods

### Ethical approval

The experiments were carried out based on the permission (No. PE/EA/1335-8/2019) of the Animal Protection Authority of the Hungarian Government Office, ensuring compliance with all relevant guidelines, regulations, and the ARRIVE guidelines.. The Hungarian Government Office is authorized to grant ethical approval in accordance with Hungarian Government Decree No. 40/2013, which is aligned with the European Union Directive 2010/63/EU. The animals were euthanized via cervical dislocation. We opted not to use sedatives prior to euthanasia, as they could potentially interfere with the inflammatory processes under study, thus potentially impacting our results. All experimental methods employed were duly approved within the scope of the Animal Experiment Allowance by the Animal Protection Authority.

### Animal model

Male BALB/C strain mice (*Mus musculus*), aged 8–10 weeks, were sourced from the National Institute of Oncology of Hungary (Budapest, Hungary) with specific pathogen-free (SPF) status. These animals were accommodated in standard housing conditions, including chip tray-lined boxes, consistent mouse feed, and ad libitum access to drinking water. The environment was regulated to maintain a temperature of 20–24 °C and a relative humidity of 60%, complemented by a 12-h light/dark cycle. Daily visual monitoring of the animals was conducted to ensure their well-being.

### ZnO fume generation and fume concentration control

In our experiments, we modeled realistic conditions using welding fumes produced via thermal methods rather than predetermined concentrations and sizes of zinc oxide (ZnO) particles. To generate fresh ZnO fumes, we employed the Tungsten Inert Gas (TIG) welding technique on analytic grade Zn shots (Merck Group, Darmstadt, Germany), using a Rehm TIGER 180 AC/DC High welding machine (Rehm GmbH., Uhingen, Germany) with 99.99% pure argon shielding gas (Linde Hungary Ltd., Répcelak, Hungary). The welding operation adhered to professional standards, setting the welding current at 80 A on a WT40 type tungsten electrode (diameter 2.4 mm) and the shielding gas flow at 5 l/min. The ZnO fume-polluted air was then circulated through the experimental setup and filtered back into the workshop's air via a Kemper Smart Master (Kemper GmbH., Vreden, Germany) fume extraction and filtering device.

The ZnO air concentration in our treatment chamber was continuously monitored using an Aeroqual Model 500 instrument (Aeroqual, Auckland, New Zealand), equipped with PM10 and PM2.5 sensors in ppm as dimension. This instrument's calibration certificate confirmed the accuracy of the measurements (Figure S1, Additional information section). We emphasize that continuous monitoring using the same instrument introduces non-differential errors. However, since the instrument has been calibrated and complies with laboratory QA/QC, the results are comparable. The target ZnO concentration was set to 2 mg/m^3^, below Hungary's workplace health limit concentration, while the limit value is 5 mg/m^3^ total dust as per 5/2020 (II. 6.) ITM legislation^51^. Our preliminary experiments determined that concentrations above 3–4 mg/m^3^ severely impair mouse health, prompting us to opt for a lower test material concentration. Aligned with the Australian workplace exposure standard, where 1 mg/m^3^ equals 0.3 ppm at 25 °C and 101.3 kPa, as per the draft evaluation report published by Safe Work Australia, the target 2 mg/m^3^ ZnO concentration equated to 0.6 ppm in our experiment^52^. Since our thermal ZnO fume production method resulted in more particle concentration than the required 0.6 ppm, we intermittently generated ZnO fumes. In each cycle, fumes were produced until the PM10 concentration reached 1.2 ppm, then allowed to drop to 0.1 ppm over time, as illustrated in Fig. 2a. This method was crucial, as it ensured that we maintained the desired concentration level while avoiding excessive exposure.

Before animal treatment, the generated ZnO fume was analyzed using SEM for particle size and morphology, and EDS for chemical composition, at the laboratory of the Material Science and Technology Department, Faculty of Mechanical Engineering, Budapest University of Technology and Economics, Hungary.

### Animal treatment

**Figure 5:**
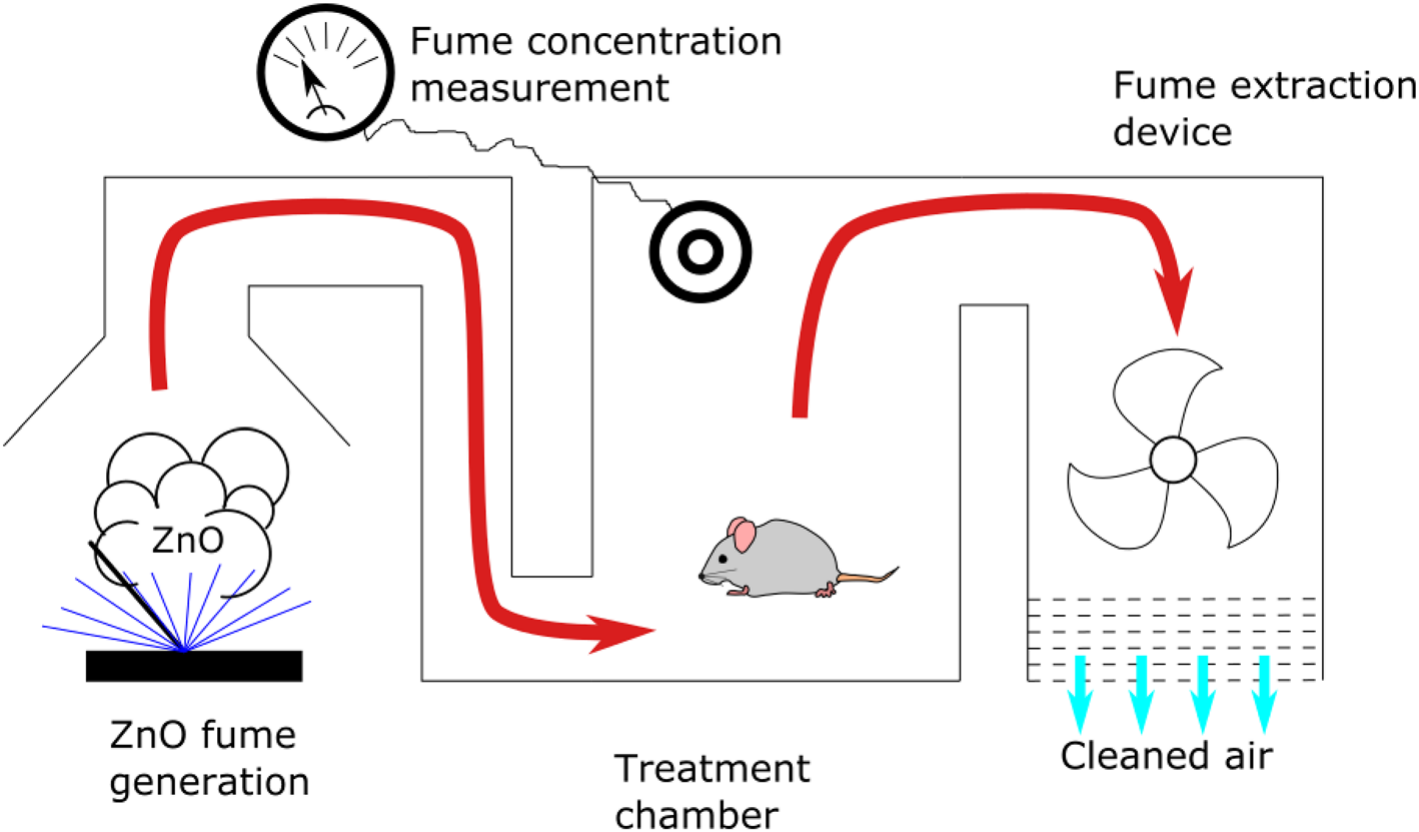
Schematic diagram of the animal treatment setup used in our experiments. The red arrow indicates the path of ZnO fume-polluted air; blue arrows represent the flow of filtered air.

As mentioned earlier, zinc oxide (ZnO) fumes were intermittently generated in a welding cabinet and then transferred to an animal treatment cabinet. Here, animals were individually housed in treatment chambers of an EMKA Whole Body Plethysmograph system (EMKA Scientific, Paris, France). Throughout the fume exposure, the animals had free access to food and water. The airflow rate in the chambers was maintained at 0.5 l/min. Each treatment session lasted for 4 h daily, preceded by an acclimatization period of about 10 min. Four animals were exposed in each treatment session, conducted over three consecutive days. The fume concentration within the animal treatment cabinet was continuously monitored, with polluted air being filtered and recirculated as previously outlined. The treatment setup is depicted in Fig. 5. Control animals were also placed in the same technical setup, but they were not exposed to any fumes during their incubation period.

### Pretreatment and selection of susceptible individuals by measuring locomotor activity

In each of our experiments, four animals were exposed to air containing ZnO fumes. Recognizing that fever in animals often leads to reduced locomotion activity, we identified decreased activity as a potential fever indicator^53^. Post-exposure, the animals were monitored for 72 h using the Teledyne FLIR Duo R thermal imaging camera (Teledyne-FLIR, Wilsonville, Oregon, USA). This camera was set up to capture thermal images of the four individually housed animals, ensuring the field of view encompassed all cages. Images were taken at 6-min intervals and analyzed using R Studio statistical software. For analysis, the images were divided into four horizontal sections, each representing one animal's cage. An R script identified the hottest points within these sections, corresponding to the animals' locations in the cages^54^. We summarized the position data in 6-h intervals to create two-dimensional frequency charts. These charts helped identify animals with limited movement, confined to a smaller area than their counterparts, while considering their circadian rhythms. Animals exhibiting low locomotor activity for at least two consecutive 6-h intervals were considered to be exhibiting signs of fever, indicative of MFF sensitivity, and were selected for further examination. This preliminary phase of the experiment was conducted three times.

### Main experiment with susceptible animals

Animals identified as sensitive during the pretreatment phase underwent the main experiment following a 3-week exposure-free rest period. This interval was crucial to minimize any potential development of tolerance. These mice were then subjected to the same experimental conditions previously described: daily 4-h treatments for three consecutive days. At 3 and 12 h post the final zinc exposure, two mice were euthanized at each time point, and samples from their lungs and mediastinal lymph nodes were collected. To preserve these samples for subsequent analysis, they were stored in RNAlater stabilization solution at room temperature (ThermoFisher Scientific, Waltham, MA, USA).

### RNA isolation

Total RNA was extracted from the collected samples using the QIAGEN RNeasy Kit, strictly adhering to the manufacturer's protocol (Qiagen, Hilden, Germany). A crucial step in our methodology was the pooling of samples collected at each time point (from both lungs and lymph nodes of the two animals) into a single sample prior to RNA isolation. The concentration of the extracted RNA was then quantified using a NanoDrop ND-1000 UV–Vis Spectrophotometer (Thermo Fisher Scientific Inc., Waltham, MA, United States).

### Transcription of RNA into copyDNA

Complementary DNA (cDNA) was synthesized from the isolated RNA utilizing the Qiagen RT2 First Strand Kit (Qiagen, Hilden, Germany). This process was conducted in strict accordance with the manufacturer's guidelines, which involved a thermal profile of 42 °C for 15 min, followed by 95 °C for 5 min.

### Gene expression measurement by quantitative real-time PCR

Real-time PCR was conducted to assess the expression of 84 genes associated with inflammatory mediators, utilizing the Qiagen RT2 Profiler PCR Array Mouse Inflammatory Cytokines and Receptors (PAMM-011ZA) kit (Qiagen, Hilden, Germany). The detailed list of these genes is available in the Additional information Table S1.

The RT-PCR reactions were carried out in a Bio-Rad CFX96 Touch Thermal Cycler (Bio-Rad Laboratories Inc., Hercules, CA, USA). Cycle threshold (Ct) values were calculated using the Bio-Rad CFX Maestro software, with gene expression values determined through ΔΔCt analysis.

Our assay incorporated internal, positive, and negative controls, alongside the evaluation of 5 housekeeping genes. The most suitable housekeeping gene for the ΔΔCt analysis was selected using the RefFinder freeware online tool (http://blooge.cn/RefFinder/).

We compared the gene expression results from the tissue samples of ZnO-exposed mice to those from lung and mediastinal lymph node samples of two untreated, healthy mice. Control animal values were standardized to 1, and a tenfold change in gene expression, either up or down compared to control values, was considered significant. This tenfold threshold was based on the manufacturer's guidelines and relevant literature^55^. Due to each gene being analyzed in a single well, a more extensive statistical analysis was not feasible.

### Supplementary Information

Supplementary Information.

## Data Availability

The dataset supporting this paper is included into the Additional information section. Further data are available from the corresponding author on reasonable request.
