# Supplementary material for: Immune response to zinc oxide inhalation in metal fume fever, and the possible role of IL-17f
Source: Sci Rep. 2023 Dec 14;13:22239. doi: 10.1038/s41598-023-49430-5 (PMC10721908; doi:10.1038/s41598-023-49430-5)
Supplement: Supplementary file 1 — Supplementary Information. [file 41598_2023_49430_MOESM1_ESM.doc]

**Additional information**

Competing interests

The authors declare no competing interests.

**Table S1.,** Gene list of Qiagen RT2 Profiler PCR Array Mouse Inflammatory Cytokines and Receptors (PAMM-011ZA) kit, according to the manufacturers’ description

|  | 1 | 2 | 3 | 4 | 5 | 6 |
| --- | --- | --- | --- | --- | --- | --- |
| A | Aimp1 | Bmp2 | Ccl1 | Ccl11 | Ccl12 | Ccl17 |
| B | Ccl4 | Ccl5 | Ccl6 | Ccl7 | Ccl8 | Ccl9 |
| C | Ccr6 | Ccr8 | Cd40lg | Csf1 | Csf2 | Csf3 |
| D | Cxcl15 | Cxcl5 | Cxcl9 | Cxcr2 | Cxcr3 | Cxcr5 |
| E | Il15 | Il16 | Il17a | Il17b | Il17f | Il1a |
| F | Il2rg | Il3 | Il33 | Il4 | Il5 | Il5ra |
| G | Nampt | Osm | Pf4 | Spp1 | Tnf | Tnfrsf11b |
| H | Actb | B2m | Gapdh | Gusb | Hsp90ab1 | MGDC |
|  |  |  |  |  |  |  |
|  | 7 | 8 | 9 | 10 | 11 | 12 |
| A | Ccl19 | Ccl2 | Ccl20 | Ccl22 | Ccl24 | Ccl3 |
| B | Ccr1 | Ccr10 | Ccr2 | Ccr3 | Ccr4 | Ccr5 |
| C | Cx3cl1 | Cxcl1 | Cxcl10 | Cxcl11 | Cxcl12 | Cxcl13 |
| D | Fasl | Ifng | Il10ra | Il10rb | Il11 | Il13 |
| E | Il1b | Il1r1 | Il1rn | Il21 | Il27 | Il2rb |
| F | Il6ra | Il6st | Il7 | Lta | Ltb | Mif |
| G | Tnfsf10 | Tnfsf11 | Tnfsf13 | Tnfsf13b | Tnfsf4 | Vegfa |
| H | RTC | RTC | RTC | PPC | PPC | PPC |

**Table S2.,** Gene expression rate results of our experiment. Data are showed as x-fold of the untreated control level, which is considered as 1-fold gene expression rate

| **Gene** | **3h gen exp** | **12h gen exp** | **Gene** | **3h gen exp** | **12h gen exp** | **Gene** | **3h gen exp** | **12h gen exp** |
| --- | --- | --- | --- | --- | --- | --- | --- | --- |
| Aimp1 | 1,63 | 1,98 | Cx3cl1 | 0,79 | 3,85 | IL-2rg | 1,37 | 1,23 |
| Bmp2 | 3,60 | 8,67 | Cxcl1 | 4,88 | 33,79 | IL-3 | 8,22 | 32,07 |
| Ccl1 | 10,17 | 34,15 | Cxcl10 | 0,94 | 16,96 | IL-33 | 3,79 | 1,54 |
| Ccl11 | 3,38 | 7,39 | Cxcl11 | 0,88 | 31,70 | IL-4 | 7,74 | 31,70 |
| Ccl12 | 8,80 | 13,97 | Cxcl12 | 0,06 | 1,95 | IL-5 | 5,87 | 20,23 |
| Ccl17 | 9,53 | 16,91 | Cxcl13 | N.a. | 5,43 | IL-5ra | 2,82 | 19,90 |
| Ccl19 | 1,41 | 13,47 | Cxcl15 | 2,89 | 1,59 | IL-6ra | 0,68 | 2,03 |
| Ccl2 | 9,18 | 38,25 | Cxcl5 | 20,10 | 60,45 | IL-6st | 0,64 | 0,83 |
| Ccl20 | 2,37 | 18,37 | Cxcl9 | 7,55 | 22,47 | IL-7 | 1,08 | 7,74 |
| Ccl22 | 0,56 | 9,90 | Cxcr2 | 1,89 | 4,67 | Lta | 0,54 | 17,57 |
| Ccl24 | 0,17 | 24,84 | Cxcr3 | 1,48 | 3,58 | Ltb | 0,04 | 1,42 |
| Ccl3 | N.a. | 13,70 | Cxcr5 | 2,25 | 7,21 | Mif | N.a. | 6,64 |
| Ccl4 | 6,46 | 20,32 | Fasl | 4,74 | 40,01 | Nampt | 1,32 | 1,13 |
| Ccl5 | 1,33 | 1,70 | IFNγ | 0,87 | 20,76 | Osm | 6,36 | 24,96 |
| Ccl6 | 0,83 | 0,61 | IL-10ra | 0,60 | 3,39 | Pf4 | 0,31 | 0,71 |
| Ccl7 | 16,48 | 23,84 | IL-10rb | 0,14 | 1,55 | Spp1 | 3,91 | 0,79 |
| Ccl8 | 3,62 | 9,55 | IL-11 | 0,42 | 24,80 | Tnf | 3,86 | 11,71 |
| Ccl9 | 4,93 | 17,84 | IL-13 | N.a. | 26,05 | Tnfrsf11b | 3,20 | 12,63 |
| Ccr1 | 1,15 | 4,32 | IL-15 | 1,81 | 5,75 | Tnfsf10 | 0,60 | 1,95 |
| Ccr10 | 0,95 | 18,71 | IL-16 | 1,69 | 2,04 | Tnfsf11 | 4,81 | 32,54 |
| Ccr2 | 0,40 | 2,40 | IL-17a | 9,80 | 43,29 | Tnfsf13 | 0,38 | 1,94 |
| Ccr3 | 0,31 | 15,23 | IL-17b | 5,28 | 25,23 | Tnfsf13b | 0,02 | 4,96 |
| Ccr4 | 0,12 | 13,43 | IL-17f | 6,65 | 91,80 | Tnfsf4 | 0,04 | 22,07 |
| Ccr5 | N.a. | 11,79 | IL-1a | 2,79 | 14,22 | Vegfa | N.a. | 0,73 |
| Ccr6 | 3,16 | 6,89 | IL-1b | 1,19 | 2,95 | Actb | 1,27 | 0,72 |
| Ccr8 | 8,37 | 29,55 | IL-1r1 | 1,36 | 3,53 | B2m | 1,00 | 1,00 |
| Cd40lg | 4,27 | 8,04 | IL-1rn | 1,17 | 5,68 | Gapdh | 2,60 | 8,08 |
| Csf1 | 1,39 | 1,70 | IL-21 | 0,11 | 30,32 | Gusb | 1,39 | 1,78 |
| Csf2 | 4,87 | 16,77 | IL-27 | 0,05 | 31,33 | Hsp90ab1 | 1,40 | 0,67 |
| Csf3 | 7,73 | 27,87 | IL-2rb | N.a. | 2,86 |  |  |  |


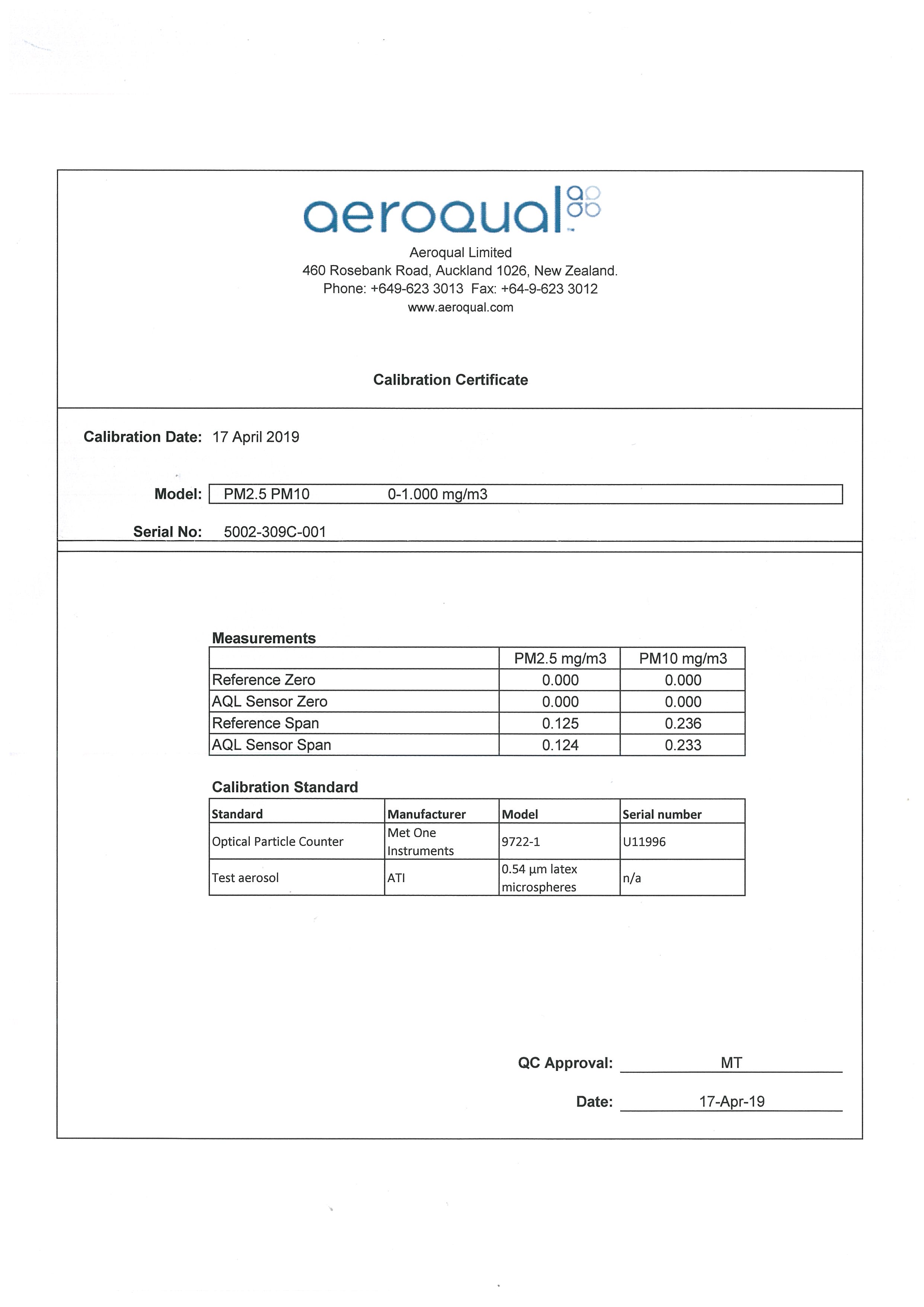


Figure S1., PM2.5 – PM10 sensor calibration certificate, received from Aeroqual Limited

Figure S2., PM10 versus PM2.5 profiles during a typical treatment regime. It is clearly visible that the PM2.5 graph is closely following the PM10 graph pattern, and comparing the PM10 and PM2.5 averages shows that that the PM2.5 particle concentrations are covering 2/3-3/4 part of the PM10 particle concentrations. Thus, most of the generated particles are small, respirable particles, that can and will reach the alveoli of the experimental animals.

Figure S2., continued. The last diagrams show the PM10 and PM2.5 measurement profiles and averages of the ambient air

Figure S3., Preliminary rtPCR results (raw ct values) of control lung (blue) and control lung + mediastinal lymph node pooled samples (dark red). It is well visible, that in case of the lung, only two cytokines (Cxcl12 and IL-21) were above the detection limit, and those also were presented in low concentrations (higher ct values, compared to the other setup) while the positive controls ct values (last three values in the graph) were nearly the same in both examinations. Taking this result into consideration, we decided to use the pooled lung-mediastinal lymph node samples in our main experiment.
